# Deficient Reporting in Avian Influenza Surveillance, Mali

**DOI:** 10.3201/eid1804.111102

**Published:** 2012-04

**Authors:** Sophie Molia, Badian Kamissoko, Maimouna Sanogo Sidibé, Adama Diakité, Mahmoudou Diall, Mamadou Racine N’Diaye

**Affiliations:** Centre de Coopération Internationale en Recherche Agronomique pour le Développement, Montpellier, France (S. Molia);; Laboratoire Central Vétérinaire, Bamako, Mali (B. Kamissoko, A. Diakité);; Direction Nationale des Services Vétérinaires, Bamako (M.S. Sidibé);; Pan African Programme for the Control of Epizootics–Mali, Bamako (M. Diall, M.R. N’Diaye)

**Keywords:** surveillance, avian influenza, highly pathogenic avian influenza virus, viruses, Africa, Mali, deficient reporting, poultry

**To the Editor:** In response to influenza outbreaks caused by highly pathogenic avian influenza virus (HPAIV) throughout western Africa as of 2006, the National Veterinary Epidemiologic Surveillance Network of Mali (EPIVET-Mali) started conducting domestic and wild bird surveillance. No HPAI outbreaks were reported to the World Organisation for Animal Health. An evaluation survey conducted in 2009 enabled identification and correction of some weaknesses in the organization and functioning of the network (*1*). However, no attempt was made to assess how much information on bird health in backyard poultry farms (which account for ≈95% of the total poultry population in Mali) actually reached EPIVET-Mali veterinarians and technicians. Therefore, we quantified reporting of clinical signs of avian diseases, especially those suggesting HPAI, by poultry owners in Mali.

We used a pilot-tested standardized quantitative and qualitative questionnaire to conduct face-to-face interviews in 32 randomly selected villages in the southern half of Mali (which accounts for 98% of the poultry population). In each village, we conducted interviews in 4 randomly chosen households. No eligibility criteria were used for household selection because all village households had poultry. Interviews were repeated 6 times (approximately every 3 months) during November 2009–February 2011 in the same villages and whenever possible in the same households. If it was not possible to repeat an interview in a previously interviewed household (absence of the household chief), the neighboring household was interviewed.

For each household, data were collected on number of sick and dead birds in the previous 3 months, clinical signs observed, and their notification or lack thereof to veterinary authorities. Households in which birds showed >3 of the following clinical signs (diarrhea, respiratory disorder, nervous signs, cyanosis of the combs or wattles, and mortality rate >50%) were considered as having clinical signs suggesting HPAI. The study was approved by the Direction Nationale des Services Vétérinaires and traditional authorities in all 32 villages, and oral consent was obtained from the poultry owners before interviews.

A total of 110–128 households were investigated at each study interval, depending on village accessibility and presence or absence of household chiefs (Table). We conducted 738 household investigations in 152 households (80 households were interviewed 6 times, 26 five times, 11 four times, 21 three times, 7 two times, and 7 one time). Observation of sick poultry in the 3 months before the interview was reported in 44.6% of household investigations, and observation of signs suggesting HPAI was reported in 12.2% (Table). Notification of veterinary authorities was reported in 13.5% of household investigations with sick poultry and in 17.4% of household investigations with signs suggesting HPAI (Table).

**Table:** Observations and reporting of sick poultry and signs of influenza suggesting HPAI virus infection, Mali, 2009–2011*

Date of investigation	Total no. households investigated	No. with sick poultry/ no. with information on sick poultry (%)	No. with HPAI signs/ no. with information on signs (%)	No. with reported sick poultry/ no. with sick poultry and information on reporting (%)	No. with reported HPAI signs/no. with signs and information on reporting (%)
2009 Nov	128	67/128 (52.3)	25/124 (20.2)	7/66 (10.6)	5/25 (20.0)
2010 Feb	128	68/128 (53.1)	21/123 (17.1)	4/68 (5.9)	0/21 (0.0)
2010 May	127	47/127 (37.0)	6/118 (5.1)	9/45 (20.0)	2/6 (33.3)
2010 Sep	110	37/110 (33.6)	17/107 (15.9)	3/34 (8.8)	2/17 (11.8)
2010 Nov	124	53/124 (42.7)	7/119 (5.9)	12/51 (23.5)	4/7 (57.1)
2011 Feb	121	57/121 (47.1)	10/115 (8.7)	8/55 (14.5)	2/10 (20.0)
Total	738	329/738 (44.6)	86/706 (12.2)	43/319 (13.5)	15/86 (17.4)

*Information was obtained for 3 months before the interview. HPAI, highly pathogenic avian influenza.

When we considered the 80 households interviewed 6 times, observation of sick poultry and signs suggesting HPAI varied over time (p = 0.043 and p = 0.018, respectively, by Cochran Q test), whereas variation over time could not be tested for notification because of an insufficient number of observations. When we considered all 738 household investigations as independent investigations, observation of sick poultry and signs suggesting HPAI varied over time (p = 0.008 and p<0.001, respectively, by χ^2^ test), but reporting of sick poultry did not vary over time (p = 0.06, by χ^2^ test). Reporting of signs of HPAI could not be tested.

These results illustrate gaps in reporting signs suggesting HPAI by backyard poultry owners. Although these signs could also be those of Newcastle disease, which is present in Mali (*2*), these signs should be reported because HPAI and Newcastle disease are officially targeted by EPIVET-Mali. One survey attempted to similarly quantify the level of HPAI reporting in Africa. In Kwara State in Nigeria, 56.5% of respondents indicated that they would not notify officials if they suspected HPAI in their flocks (*3*). Reluctance of poultry owners to comply with notification and culling obligations has also been reported in Indonesia (*4*). Several studies that assessed knowledge and practices of poultry workers with regard to avian influenza have been conducted in different countries, including developing countries (*5**,**6*). These studies were useful for better defining content of risk mitigation advice messages and the audience they should primarily target.

In our survey, occurrence of disease in Mali varied over time, which was expected because of the seasonal pattern of many avian diseases, especially Newcastle disease, in western Africa (*7*). However, reporting of sick poultry did not vary over time despite seasonality of activities in rural areas. Lack of awareness of who to report to, fatalistic attitudes toward animal diseases, and mistrust toward the government and its compensation schemes are among the major constraints affecting the likelihood of HPAI signs being reported (*3**,**6**,**8*). However, approaches associating socioanthropology and epidemiology have recently been developed to help solve the problem posed by deficient reporting (*9*).
